# The placenta: phenotypic and epigenetic modifications induced by Assisted Reproductive Technologies throughout pregnancy

**DOI:** 10.1186/s13148-015-0120-2

**Published:** 2015-08-21

**Authors:** Cécile Choux, Virginie Carmignac, Céline Bruno, Paul Sagot, Daniel Vaiman, Patricia Fauque

**Affiliations:** Hôpital de Dijon, Université de Bourgogne, Service de Gynécologie-Obstétrique, Médecine Fœtale et Stérilité Conjugale, 21079 Dijon, France; Equipe GAD, Génétique des Anomalies du Développement, EA 4271, Université de Bourgogne, Dijon, France; Hôpital de Dijon, Université de Bourgogne, Laboratoire de Biologie de la Reproduction, 21079 Dijon, France; Institut Cochin, Team Epigénétique et Physiopathologie de la Reproduction, U1016 Inserm/UMR8104 CNRS/UMR-S8104, 24, rue du Faubourg St Jacques, 75014 Paris, France

**Keywords:** Assisted Reproductive Technologies, Epigenetic, Imprinted gene, Placenta, Pregnancy

## Abstract

Today, there is growing interest in the potential epigenetic risk related to assisted reproductive technologies (ART). Much evidence in the literature supports the hypothesis that adverse pregnancy outcomes linked to ART are associated with abnormal trophoblastic invasion. The aim of this review is to investigate the relationship between epigenetic dysregulation caused by ART and subsequent placental response. The dialogue between the endometrium and the embryo is a crucial step to achieve successful trophoblastic invasion, thus ensuring a non-complicated pregnancy and healthy offspring. However, as described in this review, ART could impair both actors involved in this dialogue. First, ART may induce epigenetic defects in the conceptus by modifying the embryo environment. Second, as a result of hormone treatments, ART may impair endometrial receptivity. In some cases, it results in embryonic growth arrest but, when the development of the embryo continues, the placenta could bring adaptive responses throughout pregnancy. Amongst the different mechanisms, epigenetics, especially thanks to a finely tuned network of imprinted genes stimulated by foetal signals, may modify nutrient transfer, placental growth and vascularization. If these coping mechanisms are overwhelmed, improper maternal-foetal exchanges occur, potentially leading to adverse pregnancy outcomes such as abortion, preeclampsia or intra-uterine growth restriction. But in most cases, successful placental adaptation enables normal progress of the pregnancy. Nevertheless, the risks induced by these modifications during pregnancy are not fully understood. Metabolic diseases later in life could be exacerbated through the memory of epigenetic adaptation mechanisms established during pregnancy. Thus, more research is still needed to better understand abnormal interactions between the embryo and the milieu in artificial conditions. As trophectoderm cells are in direct contact with the environment, they deserve to be studied in more detail. The ultimate goal of these studies will be to render ART protocols safer. Optimization of the environment will be the key to improving the dialogue between the endometrium and embryo, so as to ensure that placentation after ART is similar to that following natural conception.

## Review

### Introduction

Much evidence in the literature supports the hypothesis that some adverse pregnancy outcomes observed after ART originate from suboptimal placental function caused by abnormal trophoblastic invasion. Indeed in humans, after adjusting for several confounding factors, the risk of spontaneous abortion is higher in ART cohorts than in spontaneous pregnancies [1–3]. Similarly, in several animal models, more abortions are reported after IVF, culture or superovulation than with natural conception [4–6]. Then, throughout a pregnancy following ART, placental-related defects can also occur [7]. Notably, human studies found an increased risk of gestational hypertension, preeclampsia, placenta praevia and placental abruption [7]. In addition, the risks of low birth weight [8] and prematurity [9, 8] were increased after ART. In the same way, intra-uterine growth retardation (IUGR) as well as overgrowth has been described in animals following ART procedures [10, 11, 4, 12–18]. Even if co-existing maternal risk factors (such as BMI, maternal age and infertility status) may affect placental development, the artificial manipulation of gametes and/or embryos could also play a role.

The aim of this review was to investigate the phenotypic and epigenetic mechanisms by which ART could interfere with placental formation and function, resulting in placenta-related adverse pregnancy outcomes. The first paragraph will insist on the key role of epigenetics in placental function. Then, the ART-induced placental variations occurring throughout pregnancy will be reported. To finish, the potential long-term effects of these placental modifications and the future research perspectives will be addressed.

#### Proper epigenetic regulation is essential for a functional placenta

**Fig. 1:**
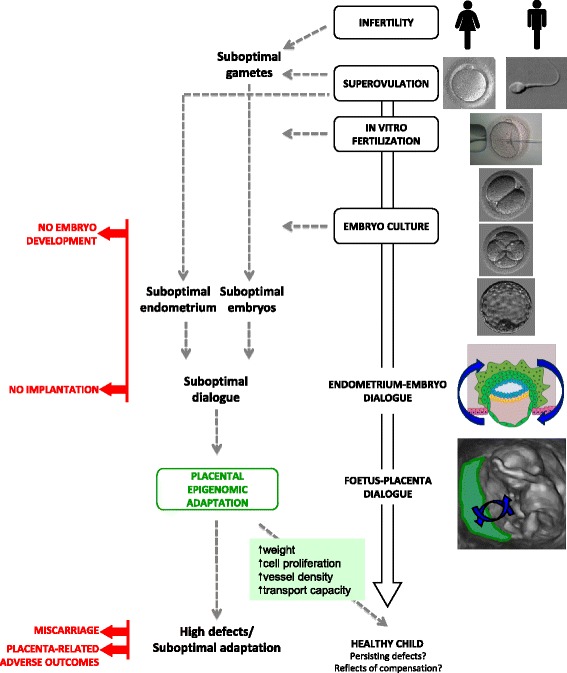
ART can impair the dialogue between the endometrium and embryo and lead to suboptimal trophoblast invasion. Infertility per se could be responsible for suboptimal gametes, and several ART steps (such as superovulation and embryo culture) may also be responsible for suboptimal embryo development, both potentially leading to embryo development arrest. In addition, superovulation may impair endometrium receptivity. Later, the placentation may be suboptimal and cause miscarriage or placenta-related adverse outcomes. However, a smart dialogue between the foetus and placenta could bring adaptive responses through regulated epigenetic mechanisms leading to increased weight, cell proliferation, increased vessel density and increased transport capacity. At birth, epigenetic variations present in cord blood or placentas could either reflect persisting variations/defects or ongoing compensation at the time of birth

1. Epigenetics in placental function
  In mammals, the placenta is a pregnancy-specific temporary organ that creates intimate contact between mother and foetus ensuring the maintenance of gestation and foetal well-being by the exchange of gases, nutrients and waste products [19]. It originates from the peripheral multipotent cells of the blastocyst (trophectoderm). In humans, placental syncytiotrophoblasts formed by the fusion of cytotrophoblasts constitute the site of exchange between the maternal and foetal circulation. It has specific endocrine functions, such as the production of placental hormones, but it also functions as a barrier, ensuring a stable environment to a foetus deprived of efficient defence mechanisms against various stresses (oxidative, xenobiotic, chemical) [20]. A finely tuned temporal and spatial regulation of trophoblastic invasion is essential for proper future function of the placenta and foetal development [21]. This involves molecular crosstalk between the endometrium and trophoblast [21].
  Notably, epigenetic regulation is a significant factor in placental development and adaptive function to environmental stress [22].
  Epigenetics may be defined as a set of cell-based molecular mechanisms able to modify gene expression. These mechanisms are heritable through mitosis or even sometimes meiosis and not sustained by DNA sequence variation [23]. Epigenetic regulation controls transcription at two levels: directly on the DNA (through DNA methylation/hydroxymethylation mechanisms) and on the proteins around which the DNA is wrapped to constitute the nucleosomes (histone modifications). Epigenetic regulation also controls translation or mRNA stability by the expression of non-coding RNAs (such as microRNA, Piwi, and Miwi).
  For instance, imprinted genes, which are epigenetically regulated, are abundantly expressed in foetal and placental tissues and are apparently absent in non-placental organisms [24, 25]. It is postulated that genomic imprinting coevolved with placentation or drove the evolution of the placenta [26], sometimes through modifications of retrotransposons [27]. Imprinted genes are expressed in a parent-of-origin manner thanks to epigenetic modifications silencing either the paternal or the maternal allele. These epigenetic modifications (DNA methylation being the most described) are established in a sex-specific manner during gametogenesis on regulatory sequences referred to as imprinting control regions (ICRs). After fertilization, these ICRs act in *cis* to achieve monoallelic expression of most imprinted genes. Up to now, approximately 150 imprinted genes have been identified in mice and humans. In mice, these are under the control of 23 identified ICRs [28–30] (http://www.geneimprint.com/site/genes-by-species). Interestingly, they are generally not imprinted in all tissues, and the imprinted pattern can be limited to a precise developmental stage. In addition, the conservation of imprinted status or even the sense of the imprinting (maternal or paternal allele expressed) may vary between mammalian species [28]. Imprinted genes, which represent a very small percentage of genes, appear to play essential roles in embryonic growth and placental development by regulating the transport capacity of the placenta thereby controlling the supply of nutrients [31, 32]. During preimplantation development, genomic imprinting is jeopardized by global DNA demethylation, and some actors such as the complex Zfp57/TRIM28/KAP1 are required to protect epigenetic imprinting marks [33]. Moreover, imprinted genes are functionally haploid by definition and thus potentially more susceptible to mutations and epimutations [34]. Their dysregulation may therefore have major consequences on the placental phenotype with long-term consequences for the developmental programming of adult health and disease [35].
2. Epigenetic modifications in the placenta and adverse pregnancy outcomes
  To function adequately, the developing placenta needs the proper epigenetic regulation of imprinted and non-imprinted genes. Indeed, experimental studies conducted in both humans and animals have clearly shown the importance of epigenetics in the regulation of placental development. For example, drug-induced disruption of DNA methylation was able to inhibit human trophoblastic invasion in vitro by disturbing the expression of epigenetically regulated genes such as E-cadherin [36] as well as the proliferation of trophoblast cells in rat placenta [37]. The deletion of placental-specific *Igf2* in mice consistently led to reduced placental growth and subsequent foetal growth restriction [38].
  In addition, numerous findings proved that disturbed placental epigenetic regulation may cause abnormal trophoblastic invasion, which may contribute to the pathophysiology of some spontaneous miscarriages, IUGR and preeclampsia.
  Indeed, in humans, *DNMT1* expression (DNA methyltransferase 1 involved in DNA methylation maintenance) and global DNA methylation were significantly lower in chorionic villi from early foetus losses than in those harvested following selective pregnancy termination [39].
  Moreover, in humans and animals, a great number of associations have been found between IUGR and epigenetic variations of imprinted or non-imprinted genes in placentas. Notably, by analyzing more than 200 human term placentas, Banister and colleagues found that the DNA methylation pattern of 22 loci was highly predictive of IUGR [40]. In mice, induced loss of imprinting and the subsequent overexpression of the imprinted *Phlda2* gene were able to trigger placental and foetal growth retardation in the offspring whereas its deletion caused overgrowth [41]. Similarly, in humans, some authors demonstrated that *PHLDA2* was up-regulated in the placenta in cases of IUGR [42–44] and that its expression level correlated negatively with birth weight [45]. As it is considered a negative growth regulator, the authors suggested that this imprinted gene potentially plays a direct role in the pathophysiology of IUGR.
  Other imprinted genes were also up-regulated (*CDKN1C*) or down-regulated (*MEG3*, *GATM*, *ZAC1*, *GNAS*, *MEST*, *IGF2*) in IUGR placentas [42, 46, 47, 44]. Some of these differential expressions were associated with decreased placental methylation, as was the case for *H19/IGF2* ICR1 [48], or loss of imprinting, as was the case for *ZAC1 (=PLAGL1)* and *H19* differentially methylated regions (DMRs) [42].
  In addition, other examples of non-imprinted genes highlight the possibility that foetal growth potential could be negatively impacted by epigenetic dysregulation in the placenta. Ruebner and colleagues pointed out that expression of Syncytin-1, a protein that promotes cellular fusion in the syncytiotrophoblast, was lower in human IUGR placentas than in controls [49]. The same team recently linked decreased expression of this protein to epigenetic hypermethylation of its promoter [50].
  In an induced IUGR rat model, Reamon-Buettner and colleagues reported decreased expression and aberrant DNA methylation patterns of the promoter region of the *Wnt2* gene, which is known to be implicated in placental vascularization [51]*.* In humans, the same pattern was found with lower *WNT2* expression and higher DNA methylation in growth-restricted neonates than in controls [52].
  Interestingly, epigenetic changes were also found on repeated sequences. For example, Michels and colleagues found an increased LINE-1 methylation level in placental tissues from low birth weight infants [53]. Other evidences about preeclampsia reinforce the idea that epigenetic disorders may be involved in abnormal trophoblastic invasion. Actually, mice with induced loss of expression of the imprinted *Cdkn1c* gene developed a preeclampsia-like syndrome, with hypertension and proteinuria [54]. Besides, widespread DNA methylation changes were found in placentas of a cohort of patients suffering from early onset preeclampsia but not in gestational age-matched controls [55]. Some of these methylation modifications correlated negatively with expressional changes, especially for genes implicated in angiogenesis (*such as EPAS 1* and *FLT I*). Moreover, *BHLHE40*, a gene coding for a protein that can prevent trophoblast differentiation exhibited significantly decreased DNA methylation and increased expression in preeclampsia placentas [55]. In addition, the expression of maspin (*SERPINB5*), a serine protease inhibitor and an inhibitor of cell migration [56], which may modify trophoblast cell invasion in the first trimester [57], could also be modified in preeclampsia. In the same family of genes, SERPIN A3 is a specific inhibitor of elastase, which plays a crucial role during the implantation process. SERPIN A3 displayed decreased methylation and increased gene expression in placentas from pregnancies complicated by preeclampsia compared with controls [58], through a complex epigenetic regulation [59]. As for IUGR, several studies highlighted the increased methylation [50] and reduced expression of *syncytin-1*, as well as the down-regulation of *WNT2* in preeclamptic placentas. These modifications were possibly responsible for impaired placental function [60]. Interestingly, epigenetic modifications could also correlate with the severity of the disease. For instance, hypertension tended to be more severe in preeclamptic women with biallelic expression of *H19*, than in women with normally imprinted expression of this gene [61]. Recently, Anton and colleagues demonstrated a correlation between disease severity and alterations in DNA methylation (hypermethylation of *CDH11*, *COL5A1*, *TNF*, hypomethylation of *NCAM1*) in preeclamptic placentas [62].
  In summary, there is a wealth of data highlighting the particular role of epigenetics in placental regulation and the potential link between epigenetic dysregulation and adverse pregnancy outcomes.
  The notion of epigenetic risk emerged in recent decades and a recent meta-analysis confirmed the increased risk of imprinting disorders (such as Beckwith-Wiedemann and Silver-Russel syndromes) after ART [63]. This raised the issue of potential methylation defects associated with ART [64]. Most studies that have examined the methylation status of imprinting genes in foetuses or placentas in animal models or in humans have associated epigenetic anomalies with adverse effects on embryonic development [65].
  What follows aims to investigate the placental modifications induced by ART and to understand their link with adverse pregnancy outcomes. The hypothesis is that epigenetic dysregulation could constitute the logical link between environmental changes due to ART, abnormal trophoblastic invasion and subsequent adverse pregnancy outcomes. Indeed, ART, via epigenetic dysregulation, could disturb the dialogue between the embryo and endometrium and cause abnormal trophoblastic invasion, which triggers placental adaptive responses (Fig. 1).

#### ART and trophoblastic invasion disturbances

1. ART and the epigenetic status of the conceptus
  In animal models (especially in mice), most studies have shown that ART procedures (such as superovulation and embryo culture), whether isolated or in association, could lead to blastocyst epigenetic defects in several loci (such as *H19*, *Snrpn*, *Peg3*, *Kcnq1ot1* genes as well as repetitive sequences) [66–71].
  Moreover, these epigenetic abnormalities were not restricted to the early stages. In mice, several studies reported placenta-specific imprinting defects after implantation, appearing in suboptimal culture conditions, such as in vitro culture associated with in vitro fertilization [5], embryo transfer [72], poorer media [73, 69] or increased oxygen concentration [74] (Table 1). When assessed by transcriptomics, it was clear that the modifications of placental gene expression in mice placenta at mid-gestation were very different depending on the richness of the culture milieu. They were much stronger when simple M16 culture medium was used than when the more complex G1/G2 medium was used [75]. Interestingly, amongst the modified genes, imprinted genes were overrepresented. Recently, Hossain and colleagues found that other aspects of epigenetics could be affected by in vitro manipulations by observing the down-regulation of miRNAs in bovine placentas from in vitro production (IVF and in vitro culture) compared with those from artificial insemination [76]. Even in human placentas, epigenetic modifications were observed. Indeed, ART was associated with lower DNA methylation levels and higher expression levels of SERPINF1 [77]. This protein is ubiquitously expressed and presents a potent anti-angiogenic activity [78]. Thus, its deregulation may detrimentally affect placentation and foetal development.

**Table 1:** Conceptuses and/or placentas in mice: resorption rate, weight, gene expression and/or DNA methylation of imprinted genes

Species	GA study	Control group	Manipulation group	RR	Weight		Gene expression		Methylation		References
					F	P	F	P	F	P	
Mouse	E14	Blastocyst transfer	SO, IVC M16 (1-cell=>blastocyst)	=	=	NA	= *Igf2*, *Grb10*, *Grb7*, *H19*	NA	= *H19*	NA	[73]
			SO, IVC M16+FCS (1-cell=>blastocyst)	↑	↓	NA	↓ *H19*, *Igf2*, *Grb7*	NA	↑ *H19*	NA	
							↑ *Grb10*				
Mouse	E18	SO, blastocyst transfer	SO, IVC (1-cell=>morula) 7 % O2, (morula=>blastocyst) 2 % O2, transfer	↑	↓	=	NA	= *Slc2a1*, *Slc2a3*, *Igf2*, *Igf2r*, *H19*	NA	NA	[186]
			SO, IVC (1-cell=>blastocyst) 7 % O2, transfer	=	=	=	NA	= *Slc2a1*, *Slc2a3*, *Igf2*, *Igf2r*, *H19*	NA	NA	
			SO, IVC (1-cell=>morula) 7 % O2, (morula=>blastocyst) 20 % O2, transfer	=	=	=	NA	= *Slc2a1*, *Slc2a3*, *Igf2*, *Igf2r*, *H19*	NA	NA	
Mouse	E12.5	SO, blastocyst transfer	SO, IVF, IVC KSOM/AA, blastocyst transfer	=	↓	=	NA	NA	NA	NA	[4]
			SO, IVF, IVC WM, blastocyst transfer	↑	↓	↓	NA	NA	NA	NA	
		SO, IVF, IVC KSOM/AA, blastocyst transfer	SO, IVF, IVC WM, blastocyst transfer	=	↓	↓	NA	NA	NA	NA	
Mouse	E15.5	SO, blastocyst transfer	SO, IVF, IVC KSOM/AA, blastocyst transfer	NA	↓	=	NA	↑ *Slc7a3*	NA	NA	[10]
								= *Igf2*, *H19*, *Glut1*, *Snat,1 Snat2*, *Snat4*			
								↓ *Glut3*			
	E18.5	SO, blastocyst transfer	SO, IVF, IVC KSOM/AA, blastocyst transfer	NA	↓	↑	NA	= *Snat1*, *Slc7a3*	NA	NA	
								↓ *Igf2*, *H19*, *Glut1*, *Glut3*, *Snat2*, *Snat4*			
Mouse	E9.5	In vivo fertilization	IVC KSOM/AA (2-cells=>blastocyst)	NA	NA	NA	Monoallelic*: H19*, *Snrpn*	Monoallelic: *H19*, *Snrpn*, *Ascl2*, *Peg3*	= *H19*, *Snrpn*	= *H19*, *Snrpn*	[69]
			IVC WM (2-cells=>blastocyst)	NA	NA	NA	Monoallelic: *H19, Snrpn*	Biallelic: *H19*, *Snrpn*, *Ascl2*, *Peg3*	= *H19*, *Snrpn*	Partial LOM: *H19*, *Snrpn*	
Mouse	E9.5	In vivo fertilization	SO, blastocyst transfer	NA	NA	NA	Monoallelic: *H19*, *Cdkn1c*, *Kcnq1*, *Ascl2*, *Zim1*, *Snrpn*, *Kcnq1ot1*, *Peg3*, *Igf2*, *Mkrn3*	Biallelic: *H19*	NA	NA	[72]
								High levels of misexpression: at least 1/8 IG			
								↑ *Ascl2*, = *H19*			
								↓ *Igf2*			
			SO, IVC KSOM/AA (2-cells=>blastocyst), blastocyst transfer	NA	NA	NA	Monoallelic: *H19, Cdkn1c, Kcnq1, Ascl2, Zim1, Kcnq1ot1, Peg3, Igf2*	Biallelic: *H19*	NA	NA	
								↑ *Ascl2*, = *H19*			
								↓ *Igf2*			
							*Low levels of misexpression: Snrpn, Mkrn3*				
Mouse	E9.5	In vivo fertilization	SO	NA	NA	NA	Monoallelic: *H19*, *Snrpn*, *Igf2*, *Kncq1ot1*	Biallelic: *H19*, *Snrpn*	NA	= *H19*, *Snrpn*	[79]
								Monoallelic: *Igf2*, *Kncq1ot1*			
							= *Igf2*				
								↑ *Igf2*			
		Blastocyst transfer	SO, blastocyst transfer	NA	NA	NA	Monoallelic: *H19*, *Snrpn*, *Igf2*	Biallelic: *H19*	NA	= *H19*, *Snrpn*	
								Monoallelic: *Snrpn, Igf2*			
							*= Igf2*	↑ *Igf2*			
Mouse	E10.5	SO, blastocyst transfer	SO, IVC (1-cell=>blastocyst) (M16 or sequential G1/G2), blastocyst transfer	↑	NA	NA	NA	↑ *H19*, *Igf2*, *Zac1*, *Slc38a4*, *Cdkn1c*, *Gtl2*, *Rian*, *Dlk1*, *Nna*t, *Peg3*	= *Igf2*, *H19*	= *H19*, *Igf2*, *Igf2r*, *Dlk1-Dio3*	[5]
								= *Igf2*r, *Grb10*			
								↓ *Dnc*, *Gatm*, *Mest*			
			SO, IVF, IVC (M16 or sequential G1/G2), blastocyst transfer	↑	NA	NA	NA	↑ *H19*, *Igf2*, *Igf2r*, *Zac1*, *Slc38a4*, *Cdkn1c*, *Gtl2*, *Rian*, *Dlk1*, *Nna*t, *Peg3*	= *Igf2*	= *H19*, *Igf2*, *Igf2r*, *Dlk1-Dio3*	
								= *Grb10*, *Mest*			
								↓ *Dnc*, *Gatm*			
Mouse	E14	In vivo fertilization	SO, IVF, IVC, blastocyst transfer	NA	NA	NA	↓ I*gf2*,	↑ *Igf2*	LOM: *H19*	LOM: *H19*	[187]
							↑ *H19*	↓ *H19*			
			SO, IVF, IVC, vitrifying/warming morula, blastocyst transfer	NA	NA	NA	↓ *Igf2*,	↑ *Igf2*	LOM: *H19*	LOM: *H19*	
							↑ *H19*				
		SO, IVF, IVC, blastocyst transfer	SO, IVF, IVC, vitrifying/warming morula, blastocyst transfer	NA	NA	NA	↑ *Igf2*,	↑ *Igf2*	LOM: *H19*	= *H19*	
							↓ *H19*	↓ *H19*			
Mouse	E10.5	In vivo fertilization	SO, IVF, IVC KSOM/AA 5 % O2, morula/blastocyst transfer	NA	NA	NA	Monoallelic: *Igf2, Cdkn1c, Snrpn, Kcnq1ot1*	Biallelic: *H19, Snrpn*, *Peg3*, *Cdkn1c*	= *H19, Snrpn, Peg1, Kcnq1ot1, Dlk1/Gtl2, Peg3*	= *Snrpn, Kcnq1ot1, Peg1, Dlk1/Gtl2, Peg3*	[74]
							Biallelic: *H19*, *Peg3*	Monoallelic: *Kcnq1ot1*			
										↓ *H19*	
			SO, IVF, IVC KSOM/AA 20 % O2, morula/blastocyst transfer	NA	NA	NA	Monoallelic: *Igf2*, *Snrpn*, *Kcnq1ot1*, C*dkn1c*	Biallelic: *H19*, *Snrpn*, *Peg3*, *Cdkn1c*, *Kcnq1ot1*	= *H19, Snrpn*, *Peg1*, *Kcnq1ot1*, *Dlk1/Gtl2*, *Peg3*	= *Snrpn*, *Kcnq1ot1*, *Peg1*, *Dlk1/Gtl2*	
							Biallelic: *H19*, *Peg3*				
										↓ *Peg3*, *H19*	

*E* embryonic day, *F* foetus, *FCS* foetal calf serum, *GA* gestational age, *ICSI* intra-cytoplasmic sperm injection, *IVC* in vitro culture, *IVF* in vitro fertilization, *IVPS* in vitro produced with serum, *KSOM/AA* optimal potassium-modified, simplex optimized medium with amino acids, *LOM* loss of methylation, *NA* not analyzed, *OVM* oocyte in vitro maturation, *RR* resorption rate, *P* placenta, *SO* superovulation, *SOF* synthetic oviductal fluid, *mSOF* modified synthetic oviductal serum fluid medium without serum or coculture, *WM* Whitten’s medium, ↑: increased, ↓: decreased, =: no significant difference compared with control
  Surprisingly, placenta appears to be more susceptible to modifications in DNA methylation and/or expression of imprinted genes at mid-gestation [74, 79, 69, 72] (Table 1). Discussing this observation, Mann and colleagues proposed two scenarios to explain why the defects were apparently restricted to the trophectoderm lineage [69]. In the first hypothesis, extra-embryonic cells, in contact with the culture medium, are more severely affected by in vitro culture, which is responsible for a loss of imprinting in mid-gestation placentas. Indeed, trophectoderm (TE) cells are directly exposed to the environment. Besides, they are also the first lineage to differentiate in the embryo as trophectoderm stem cells, from which the different cell lines of the future placenta will originate [80]. Other studies are in accordance with this hypothesis. Notably, TE cells from blastocysts cultured in vitro showed strong expressional modifications with the activation of stress-related pathways and the down-regulation of genes involved in placentation [81, 82]. Specifically, *Igf2* expression in TE cells was lower after IVF than in controls [81]. In the second hypothesis developed by Mann and colleagues, the embryo could be able to restore a correct imprint thanks to lineage-restricted de novo methylation occurring in inner cell mass (ICM) but not in TE cells.
  A third hypothesis involves the selection of viable embryos through active selective elimination mechanisms that act to discard embryos with abnormal imprinting before mid-gestation. Indeed, the studied embryos were those that reached this developmental stage. In mice, following ART, an increased number of resorption sites was observed. This number was even higher when the embryos were fertilized and cultured in vitro than when only cultured in vitro. This could indicate that embryos with defective imprinting do not survive and that the effect is cumulative [5, 4]. Reinforcing this idea, Yin et al. showed that mice injected with an inhibitor of DNA methyltransferase 1 (Dnmt1), an enzyme responsible for methylation maintenance, had a smaller number of implanted embryos [39]. Furthermore, at mid-gestation, these embryos had a lower global DNA methylation level, which was associated with growth retardation [39]. These results strengthen earlier experimental studies in mice that highlighted the fundamental contribution of DNA methylation enzymes to embryonic development [83].
  In summary, these data support the hypothesis that a suboptimal embryo environment induced by ART greatly disturbs the epigenetic status of not only the embryo (eventually causing development arrest) but also the extra-embryonic tissues.
2. ART and endometrial receptivity
  Apart from modifying the epigenetic status of the conceptus, another way in which ART could alter trophoblastic invasion could be its effect on the endometrium.
  Much evidence has linked poor endometrium quality to abnormal early placentation. Even though some genetic causes of endometrial defects leading to recurrent miscarriages have been described [84–86], ovarian stimulation, which is required in most ART procedures, may also be responsible for poorer endometrium quality. Since the ovary and the uterus share several signalling pathways, and since hormones secreted by the ovary have a direct effect on uterus function, ovarian stimulation probably modifies the uterine environment. This is assessed by studies that demonstrated differential expression of genes in the endometrium between stimulated and natural cycles, with a dose-response effect [87, 88].
  In mice, the implantation rate was lower and post-implantation foetal mortality was higher in superovulated recipients than in non-stimulated controls [89]. Similar observations were also reported in humans, with a dose-dependent effect: the risk of spontaneous abortion was significantly higher in women stimulated with high levels of hormones than in those stimulated with lower levels [3]. Besides, high serum estradiol levels at ovulation triggering after controlled ovarian stimulation are associated with placenta-related adverse pregnancy outcomes such as growth restriction or preeclampsia [90, 91].
  Other evidences highlight the impact of a suboptimal endometrium induced by ovarian stimulation on placental and foetal growth. Notably, hormones are known to modify birth weight. Indeed, singletons born after IVF have on average a lower birth weight than singletons born after natural cycles with mild stimulation [92]. Moreover, an inverse correlation between birth weight and estradiol levels achieved in case of IVF [93] was found. In mice, the mean weight of foetuses was also lower in stimulated than in non-stimulated recipients [89, 94].
  Surprisingly, birth weight was higher in ART-offspring after the transfer of cryopreserved/thawed embryos than with fresh embryos [95, 96]. While it could be hypothesized that this was caused by a direct effect on the embryo, differences in hormonal treatment between the two groups could have an important effect as well. In the first case (cryopreserved embryos), women are not treated with follicle-stimulating hormone (FSH) to induce multifollicular growth, while they are treated in the second case. In natural conception, when two children from the same mother are compared, the second one is usually heavier [97]. However, when the first is born following transfer of a frozen embryo and the second after IVF, the situation is reversed [95]. On average, birth weight following frozen embryo transfer is the same as that following natural conception [98]. The fact that frozen embryos are transferred without controlled ovarian hyperstimulation suggests that the endometrium-embryo dialogue is in this situation closer to the “natural” dialogue and enables normal placentation. It is also possible that freezing selects embryos with normal epigenetic profiles, by unknown putative mechanisms. However, recently, two different teams highlighted that the risk of large for gestational age and preeclampsia could be increased in frozen embryo cycles compared with fresh cycles or natural conception [99, 100]. Therefore, further studies are needed to determine the impact of the different protocols used in frozen embryo transfer (hormonal treatments used, duration of culture, cryoprotectants, culture media, etc.).
  Other data are in keeping with the hypothesis that superovulation and hormone treatment may impair placentation. For example, a recent study examining near-term placentas in superovulated mouse recipients found altered trophoblast differentiation causing a reduced maternal-foetal exchange area [94]. Besides, in humans, pregnancy-associated plasma protein A (PAPP-A) levels in maternal serum were decreased in first-trimester ART pregnancies [101–105]. PAPP-A is known to play a critical role in trophoblastic invasion [106] by contributing to maternal tolerance towards the foetus [107]. Giorgetti et al. confirmed these low levels after ART and further added that maternal serum PAPP-A levels correlated strongly and inversely with estradiol levels at ovulation triggering [108]. Accordingly, PAPP-A values were lower after the transfer of fresh embryos (when ovarian stimulation was used) than after the transfer of frozen embryos or after unstimulated cycles [101, 109].
  All these findings highlight a tight relationship between high hormone levels and impaired trophoblastic invasion presumably through decreased endometrium receptivity. Exposing the endometrium to high levels of estradiol and progesterone produced by multiple corpora lutea could possibly render it less efficient for embryo implantation than it is during natural cycles [110]. Thus, ART processes, and especially hormone treatments, may increase the rate of adverse pregnancy outcomes by inducing more trophoblastic invasion defects.
  In addition to hormone treatments, infertility per se could involve an altered uterine environment. For example, some authors recently suggested that endometriosis may be accompanied by epigenetic modifications implicated in diminished endometrial receptivity and altered gene expression. Epigenetic modifications on the promoter of a mediator of endometrial receptivity, HOXA10, may be one of the mechanisms involved, as reported in women [111–113] and in several animal models [114, 115].
  To summarize, ART, through its negative effect on the endometrium-embryo dialogue, could participate in preventing successful trophoblastic invasion. This could potentially explain the occurrence of adverse pregnancy outcomes after ART. Depending on the severity of the defects, ART could gradually lead to developmental arrest, miscarriages, preeclampsia or IUGR (Fig. 1). But in most cases, pregnancies obtained after ART are able to continue without obvious immediate adverse outcomes. This sustains the hypothesis that initial defective trophoblastic invasion could trigger placental adaptive responses during pregnancy.

#### ART and the possible induction of placental adaptive responses

Nuclear transplantation in animals is known to produce placental phenotypic modifications (such as placentomegaly), to modify placental metabolism and to disturb imprinted gene expression [116, 117]. Given these placental modifications after somatic cell nuclear transfer, we wondered whether ART could trigger placental responses.

1. Phenotypic placental responses
  In the literature, several studies in animals showed that a suboptimal placenta is created by in vitro conditions but that counterbalancing mechanisms also occurred. First, a smaller quantity of TE cells developed in mouse blastocysts from in vitro culture than in naturally conceived blastocysts [82]. At later stages (12.5 dpc), IVF embryos and placentas were smaller than those in the control group [4] (Table 1). However, the placenta was slightly larger (+9 %) at 15.5 dpc and to an even greater extent (+25 %) at 18.5 dpc, while foetus weight was 16 % lower at 15.5 dpc but only 9 % lower at 18.5 dpc in the IVF group than in controls [10] (Table 1). At this later stage, cell proliferation was greater in IVF placentas than in controls, in both the labyrinth and spongiotrophoblast layers. By birth, IVF foetuses had reached the same weight as the controls [10]. In the in vitro context, placentas were found to be lighter than control placentas at early gestation and heavier at late gestation. While a larger placenta is not necessarily synonymous of a higher efficiency in nutrient and oxygen transfer, it can in this case probably contribute to a compensatory growth of the foetus, despite initial functional limitations. Similar results were observed in the sheep model: foetuses from in vitro cultured embryos were 60 % smaller than naturally conceived foetuses at day 24 of gestation, whereas no difference was found at later stages [16].
  Likewise, in humans, the enlargement of placentas has been observed in complicated pregnancies associated with low birth weight, such as pregnancies with late-onset preeclampsia, foetal death or advanced maternal age [118–120]. Interestingly, the same phenomenon was seen in singletons from ART. Placentas from ART pregnancies were overrepresented in the highest quartile of weight, and the placental weight/birth weight ratio was commonly higher, while the mean birth weight was lower, even after adjusting for potential confounding factors [121]. This increased placental weight after IVF could be the result of compensatory responses.
2. Mechanisms involved in placental responses
  Metabolic pathways
    According to Coan and co-workers, the placental phenotype is responsive to nutritional conditions. When foetal nutrient availability is compromised, it adapts to maximize the nutrient transfer capacity [122]. These compensatory mechanisms may start from the blastocyst stage, within extra-embryonic lineages. Actually, using a mouse maternal protein restriction model, some authors demonstrated increased endocytosis, cell proliferation and invasiveness in the trophectoderm, which may reveal enhanced nutrient capture [123, 124]. The up-regulated expression of nutrient supply genes such as glucose and system A amino acid transporters was shown in small murine placentas during late gestation, thus reflecting a response to foetal demand signals [122]. The foetus itself plays a role in its own development and growth by sending signals to the placenta, which will respond by regulating genes involved in growth control, specific transport systems and vascularization [125].
    These metabolic responses are well-illustrated in IVF studies on animal species [126, 15, 127]. Indeed, at early gestation, bovine conceptuses after IVF and culture displayed placentas with decreased blood vessel density, while at late gestation, placentas had greater blood vessel density [15, 127]. This impaired placental vasculogenesis early in gestation was also reported for sheep embryos developed in vitro [128]. This compensatory process could implicate the angiogenic pathway and particularly an angiogenic transcription factor, peroxisome proliferator-activated receptor gamma (PPARƳ) protein, which could modulate the density of maternal blood vessels throughout pregnancy [15]. In addition to the gain in vascularization, increasing cell fusion could improve foeto-maternal exchanges. Indeed, two proteins involved in membrane fusion, annexin A3 and α-SNAP, were found to be up-regulated in human term placentas obtained after ART [19].
    Besides, in human placentas after ART, genome-wide mRNA expression revealed the overexpression of genes involved in metabolism, immune response, transmembrane signalling and cell cycle control [129, 130]. Similarly, transcriptomic data in mouse placental tissues show that IVF techniques trigger the induction of genes involved in cellular proliferation and cell cycle pathways [75].
    In summary, the kinetics of placental and foetal growth altered by ART may be linked to modifications in various biological pathways, probably triggering the placental compensation phenomenon. While the complete picture of the systems that regulate this compensation is still blurred, epigenetic changes certainly play a part in the adaptive mechanisms.
  Imprinted gene network
    Concerning the regulation of this placental response, one interesting hypothesis is that potential primary dysfunctions of the placenta could be corrected by the imprinted gene network of placental mammals (IGN). The modulation of this network of coordinated imprinted genes (and probably non-imprinted genes), which are involved in growth control and specific placental transport systems, could contribute to the tight regulation of foetal growth during post-implantation development. This was described in mice for *Igf2*, *Zac1* and *H19* [131, 132] and recently in the human placenta for *ZAC1* [133].
    To support this hypothesis, in mouse placentas after ART, most genes of the IGN were up-regulated in a coordinated fashion, when compared with the control group [5]. The fact that these genes with placental reciprocal functions were up-regulated after ART despite phenotypically and morphologically normal embryos suggests that placental IGN may participate in the control of normal foetal growth in ART pregnancies. However, the methylation status of their DMRs after ART was either similar to that in controls or only slightly modified during gestation [5, 69]. In the same way, the methylation of repeated sequences (ALUYb8, α-satellites and LINE-1) were reported to be unchanged after ART [134]. Other epigenetic mechanisms, such as histone modifications, could therefore be involved. In fact, according to Lewis et al., an ancestral imprinting mechanism, restricted to the placenta, is based on histone modifications [135], which may confer the short-term and flexible response implicated in development [136–138].
    Regrettably, no evidence is available in animals at birth concerning the occurrence of epigenetic modifications in the placenta. In humans, nothing is certain (Table 2). Three studies that carried out large DNA methylation analyses using arrays found conflicting data. Indeed, the first published study described quantitative differences in global DNA methylation (briefly with a higher and a lower degree of DNA methylation in post-IVF cord blood and placental samples, respectively) and for several imprinted genes [77] (Table 2). In contrast, two recent studies reported either opposite cord blood findings [139] or none variability in DNA methylation at 25 imprinted DMRs [134] (Table 2). However, the three studies are not comparable regarding the sample size (10 individuals versus 73), the mode of reproductive treatment (IVF versus unspecified ART) and the method used.

**Table 2:** Effects of ART on imprinted genes and retrotransposable element expression and methylation in chorionic villous samples from abortion, peripheral blood, cord blood and placenta

Control group	Manipulation group	Gene	Sample	Technique for expression	Results of expression analysis		Technique for methylation	Results of methylation analysis		References
					Trends	Fold change		Trends	Differential methylation level	
30 NC	18 IVF or ICSI	*KCNQ1OT1*	CPB	NA			MS-PCR	MS-PCR: hypoM (3/12)		[188]
							MSED-qPCR	MSED-qPCR: =		
			CB					=		
			P					=		
13 NC	10 IVF	*MEST*	CB	RT-qPCR	=		Methylation array	?	21.8 %	[77]
		*SLC22A2*	CB		=			↓	3.0 %	
		*PEG10*	CB		=			↓	4.2 %	
		*PEG3*	CB		=			↓	5.2 %	
		*GNAS*	CB		=			↓	3.0 %	
		*NNAT*	CB		=			↓	1.6 %	
		*PEG3*	P		=			↑	6.7 %	
		*MEST*	P		↑	2.09-fold		↓	1.9 %	
		*SLC22A2*	P		=			↓	7.3 %	
77 NC	35 IVF	*MEST*	MPB/CB	NA			SIRPH	↑	MBP: 2.0 %, CB: 3.0 %	[143]
		*MEST*	ACM					=		
		*KCNQ1OT1*, *H19*, *SNRPN*, *GRB10*, *DLK1/MEG3 IG-DMR*, *GNAS NESP55*, *GNAS NESPas*, *GNAS XL-alpha-s*, *GNAS Ex1A*	MPB/CB					=		
	77 ICSI	*MEST*, *KCNQ1OT1*, *H19*, *SNRPN*, *GRB10*, *DLK1/MEG3 IG-DMR*, *GNAS NESP55*, *GNAS NESPas*, *GNAS XL-alpha-s*, *GNAS Ex1A*	MPB/CB/ACM					=		
77 ICSI	35 IVF	*MEST*	MPB/CB					↑	MBP: 3.0 %, CB: 3.0 %	
		*MEST*	ACM					=		
		*KCNQ1OT1*, *H19*, *SNRPN*, *GRB10*, *DLK1/MEG3 IG-DMR*, *GNAS NESP55*, *GNAS NESPas*, *GNAS XL-alpha-s*, *GNAS Exon1A*	MPB/CB					=		
29 NC	24 IVF, 14 ICSI, 4 IVF or ICSI	*KCNQ1OT1*	CVS	NA			Bisulphite pyrosequencing	↓	4.0 %	[146]
		*H19*, *MEG3*, *MEST*, *NESP55*, *PEG3*, *SNRPN*	CVS					=		
12 NC	45 ART	*H19*	CB	RT-qPCR	=		Parental allele-specific methylation	=		[145]
		*IGF2R*	CB		↓	0.61-fold		=		
		*H19*	P		↓	0.72-fold		↑ LOI		
		*IGF2*	P		↓	0.52-fold		NA		
		*IGF2R*	P		=			=		
12 NC	32 IVF, 45 ICSI	*H19*	P	NA			MS-SNuPE	=		[141]
30 NC	61 ART	*H19*	CB	NA			COBRA + sequencing	=		[140]
59 NC	59 IVF	*KCNQ1*	CB	NA^a^			Bisulfite pyrosequencing	↑	0.6 %	[142]
		*MEST*, *GRB10*, *H19*, *IGF2 DMR0*, *SNRPN*	CB					=		
		*SNRPN*	P					↑	1.7 %	
		*MEST*	P					↓	3.4 %	
		*H19*	P					↓	1.3 %	
		*GRB10*, *IGF2 DMR0*, *KCNQ1*	P					=		
27 NC	27 OI	*KCNQ1*	CB					↑	1.3 %	
		*SNRPN*	CB					↑	2.1 %	
		*GRB10*, *MEST*, *H19*, *IGF2DMR0*	CB					=		
		*SNRPN*	P					↑	2.1 %	
		*H19*	P					↓	4.5 %	
		*KCNQ1, GRB10, MEST, IGF2 DMR0*	P					=		
35 NC	5 IVF, 30 ICSI	*MEST*	P	RT-qPCR	=		Bisulfite pyrosequencing	↓	ND	[144]
		*MEG3*	P		NA			↓	ND	
		*H19*	P		↑	1.3-fold		↓	ND	
								(*H19* CTCF6)		
		*IGF2*	P		=			NA		
		*PEG3*, *SNRPN*, *KCNQ1OT1*, *IG-DMR*	P		NA			=		
121 NC	73 ART	ALU-Yb8, LINE-1	P/CB	NA	NA		Bisulfite pyrosequencing	=		[134]
		*DIRAS3*, *NAP1L5*, *ZAC1*, *IGF2R*, *FAM50B*, *MEST*, *GRB10*, *PEG10*, *PEG13*, *INPP5Fv2*, *H19*, *KCNQ1OT1*, *RB1*, *MEG3*, *SNRPN*, *ZNF597*, *ZNF331*, *C19MC*, *PEG3*, *MCTS2*, *NNAT*, *L3MTBL*, *NESP*, *GNAS XL*, *GNAS Ex1A*	P/CB				Methylation array	=		
23 NC	73 ART	*PHLDA2*, *GTL2*, *H19*, *ZNF331*, *ZNF597*, *C19MC*, *FAM50B*, *MEST*, *HYMAI*, *ZAC1*, *IGF2*, *KCNQ1OT1*	P	Sequenom iPLEX assay	Monoallelic					
8 NC	10 IVF	*GNAS* (2 sites*), PLAGL1*, *ZIM2*, *DIRAS3*	CB				Methylation array	↑	ND	[139]

*ACM* amnion/chorion membranes, *ART* assisted reproductive technologies, *CB* cord blood, *COBRA* combined bisulfite restriction analysis, *CPB* child peripheral blood, *CVS* chorionic villous samples, *hypoM* hypomethylation, *ICSI* intra-cytoplasmic sperm injection, *IVF* in vitro fertilization, *LOI* loss of imprinting, *MPB* maternal peripheral blood, *MSED-qPCR* methylation-sensitive enzymatic digestion associated with quantitative PCR method, *MS-PCR* methylation-specific PCR, *MS-SNuPE* methylation-sensitive single nucleotide primer extension, *NA* not analyzed, *NC* naturally conceived, *ND* not documented, *OI* ovulation induction, *P* placenta, *RT-qPCR* quantitative reverse transcription PCR, *SIRPH* single nucleotide primer extension assays in combination with ion pair reverse phase high performance liquid chromatography separation techniques, ↑: increased, ↓: decreased , =: no significant difference compared with control

^a^Analysed only on a subset of individuals with outrange methylation levels for three imprinted genes (*H19*, *KCNQ1*, *SNRPN*) but no comparisons between conception groups
    Moreover, other studies focusing on the DNA methylation of specific imprinted genes also generated contradictory results. Indeed, although some authors reported no epigenetic changes after ART [140, 141], several authors reported variations in methylation levels in both cord blood and/or placentas for a number of imprinted genes such as *MEST* [142, 143], *H19* [144, 142, 145], *KCNQ1OT1* [146] or *SNRPN* [142]. However, none of them agreed on the changes in DNA methylation and these variations were mild (from 0.6 to 4.5 % differential methylation levels) (Table 2). Once again, these studies are difficult to compare given the limitations similar to those mentioned above.
    However, most studies focused on normal pregnancy, thus excluding placenta-related adverse pregnancy outcomes (such as preeclampsia, hypertension, some IUGR) and therefore possibly ignoring major differences.
    Concerning the expression analysis of imprinted genes, conflicting results were also reported. Dysregulation mainly took place in the placenta and only for three imprinted genes (*H19*, *IGF2*, *MEST*) [77, 144, 145] (Table 2).
    Finally, these minimal expressional changes at term compared with more significant changes during pregnancy in animals could reflect the remains of defects that were partially compensated during prenatal life or even methylation allelic polymorphisms (placental epipolymorphism [147]).
    Thus, epigenetic “defects” in animals’ placentas after in vitro manipulations are found in most studies. Most authors consider this variety of placental phenotypes triggered by ART to originate from epigenetic errors at imprinted genes [74], but should we really consider these epigenetic modifications as “errors” or should we regard them as smart adaptation mechanisms developed by the placenta? From the results above, we can postulate that these “defects” are not all harmful for the embryo and that some could be considered compensatory mechanisms. Indeed, they reflect the balance between members of the IGN in the placenta. Biallelic expression as well as the loss of imprinting of parts of the IGN in the placenta could constitute a major compensatory mechanism to allow the developing foetus to cope with a changing or adverse environment. In response to certain stress factors that modify the early environment of the embryo, the placenta could amplify these compensatory mechanisms up to a certain point. In most cases, efficient compensation ensures normal foetal growth up to term. When the compensation is unbalanced, compensation fails and pathological features such as miscarriages, low birth weight or preeclampsia could occur. However, what remains to be determined is whether this compensation step per se could be a risk factor for certain diseases later in life.

#### Potential long-term effects of ART-related compensation during pregnancy

These modified maternal-foetal interactions, here after ART, might have consequences for outcomes in infancy and even in adulthood, especially by inducing metabolic and cardiovascular conditions [148–152]. For instance, in humans, new-borns that are either too small or too big may be vulnerable to heart disease, hypertension, type II diabetes and obesity [153–155]. In addition, the size and shape of the placenta have been related to life expectancy in men [156] and their risk for coronary heart disease [157]. Similarly, a high placenta/foetus weight ratio, considered a marker of intra-uterine stress, has been associated with hypertension later in life [158].

As mentioned above, these phenotype modifications of the foetus and placenta are found in ART pregnancies. Thus, the modified intra-uterine environment after ART may be one cause of late-onset diseases [159]. Indeed, although the majority of ART children are healthy, the available data about long-term follow-up of ART children revealed cardiovascular and metabolic risk factors [159]. Notably, children born after ART may exhibit increases in peripheral adipose tissue mass, in systolic and diastolic blood pressure, in fasting glucose levels and IGF-I and IGF-II levels as well as changes in the lipid profile [160–164]. In addition, transcriptomic data at birth revealed activation of metabolic pathways implicated in chronic disorders such as obesity and type II diabetes [77]. However, further large longitudinal studies are needed to confirm these poor outcomes.

Portha and colleagues proposed that the link between the prenatal environment and adverse long-term effects could be written through epigenetic modifications of the conceptus. These plastic responses to the early environment could be kept in memory throughout life, due to epigenetic changes such as DNA methylation and histone modifications [165]. We can postulate that ART could trigger similar processes.

Nevertheless, in humans, there is no evidence of epigenetic changes persisting into childhood. Indeed, in children conceived after IVF, reassuring data have been reported for DNA methylation for four imprinted genes and even on a global scale [166, 167]. Only one recent study observed that some epigenetic errors can still be observed during childhood, though this concerned only the imprinted gene *SNRPN* [168] whose DNA methylation levels were not found to be modified at birth after ART in either cord blood or in the placenta. However, the heterogeneity of biological samples (blood or buccal cells), age range, type of reproductive technique and the analysis of methylation could hide potential underlying differences.

Another hypothesis might reside in tissue-specific epigenetic modifications. This could explain the absence of DNA methylation variations in blood and buccal cells. Therefore, studying other tissues may reveal defects linked to specific metabolic conditions. Notably, Scherrer’s team found increased DNA methylation on the promoter of the gene encoding eNOS (NO synthase) in vascular tissues in mice obtained after ART. This resulted in reduced plasma NO concentrations, increased blood pressure and a shorter lifespan [169].

It is also interesting to consider that tissue-specific epimutations for *H19*, *Snrpn* and *Peg3* genes were described in individual mice generated by ART (ICSI or superovulation) [170].

#### Ways for medical improvement and future research

##### Ways to improve actual ART protocols

Finally, as placental defects seem to originate from an altered endometrium-embryo dialogue, optimization of the environment during ART is a cornerstone and may improve early placentation. Hence, several simple and practical improvements can be proposed. First, it is possible to optimize the quality of oocytes and the endometrial milieu by using lower doses of hormones. Second, the culture media must be optimized to limit trophectoderm cell stress. Even though the parameters of this optimization are far from being mastered, it has been clearly shown that specific culture media generate a lower degree of stress for the embryo [5, 70]. Third, the embryo and endometrium should be better synchronized either by transferring blastocyst-stage embryos (even if extended embryo culture may have per se an impact the epigenetic regulation) and/or by developing molecular diagnostic tests (for example transcriptomic, lipidomic and proteomic profiles) to assess the quality of the endometrium in order to target the best timing of endometrial receptivity [171]. Fourth, another practice recently developed by some teams, could be to freeze all embryos and transfer them during subsequent cycles with an optimally prepared endometrium [172]. However, the endometrial tests and the fourth solution need to add an embryo cryopreservation step. Recent data reported poorer obstetrical outcomes after frozen embryo cycles (reported above) and a potential negative impact of the cryopreservation itself on the regulation of DNA methyltransferases in preimplantation frozen/thawed embryos [173]. Thus, further studies are required before these strategies can be applied safely.

Another way to improve the chances of success could be post-natal correction. Since imprinted genes in the placenta appear to be major operators in regulating foetal growth, further research is needed to better understand the link they may have with future disease. All in all, imprinted genes could eventually be used as sensors to predict and better prevent diseases later in life. Interestingly, some studies suggest that customized interventions might be implemented to correct effects on phenotypic changes [153]. One example is the post-natal administration of leptin in rats, which was able to reverse the adverse effects of mother-undernutrition: the offspring phenotype as well as the expression and methylation of several hepatic genes were corrected [174]. One other example is the post-natal administration of butyrate (histone deacetylase inhibitor) in the mouse model, which normalized both DNA methylation of the promoter of the *eNOS* gene and vascular function [169]. Further studies in animals are needed to better understand tissue-specific epigenetic regulation in ART. Thus, screening for epigenetic markers during early life could be used to identify more vulnerable patients and to define an appropriate treatment to potentially correct various epigenetic defects.

#### Future research to assess the impact of ART on health

More research is needed to better understand the disturbed interactions between the embryo and the milieu, especially in artificial conditions. New insights about the regulation of actors involved in the protection/maintenance of DNA methylation at imprinted genes in a context of ART are now necessary [33]. Moreover, to our knowledge, epigenetic defects have not been studied separately in TE and ICM cells so far. Nonetheless, knowing whether epigenetic dysregulation occurs in all blastocyst cells or only in TE cells could lead to better understanding of the mechanisms implicated in placental defects caused by ART. Knowledge of such mechanisms would be important to evaluate possible consequences for the developing individual soon after birth or even later in life.

Furthermore, although placental compensation enables mice to reach a normal birth weight [10], evidence in humans shows that ART pregnancies still carry a higher risk of placenta-related adverse pregnancy outcomes [7]. These differences may stem from overwhelmed compensation mechanisms, which, in certain cases, are not fully successful. Several potential reasons may explain this limited correction in humans as compared with mice. First, although placentation is haemochorial in both humans and mice [175, 176], their placentas are not organized in the same way (labyrinth and spongiotrophoblast in mice versus villous trophoblast in humans) and differ in their morphogenesis and exchange functions [175, 177]. Second, in human ART, the cumulative effects are possibly at their utmost point because the standard method is to transfer fresh embryos from a superovulated cycle, which is not performed in mice because pseudopregnant females are used. The effects observed in animal models are therefore possibly exacerbated in humans. Third, contrary to animal models, parental infertility is the major reason why ART is used in humans, and this infertility may be partly responsible for the epigenetic disorders and abnormal placentation leading to maternal pathologies, such as abruptio placentae and preeclampsia [178–180]. Therefore, any extrapolation of animal studies to humans should be done with caution.

Moreover, concerning the methodology, most epigenetic studies have addressed the effects of ART stressors on DNA methylation at the individual gene level and often analyze one or few CpG. Thus, genome-wide as well as gene-specific approaches that can target regulatory regions (promoters, enhancers, gene body, or elsewhere) and assess functional significance is now needed. High-throughput tools, which are becoming available, may be applied more widely to study the epigenomic changes associated with ART. Otherwise, in most studies, only overall expression and methylation levels are examined (Tables 1 and 2). Although it could be valuable, monoallelic expression of imprinted genes is hard to perform, given the need for informative SNPs in parents to perform this analysis.

From a global DNA methylation point of view, placenta tissue has been shown to display a very low DNA methylation profile compared with other somatic tissues [181]. More recently, human studies on placenta samples using high-throughput tools (methylome) revealed that placenta presents large partially methylated domains (PMD) which are stable during pregnancy [182]. This unique property of the placenta might contribute to the regulation of the expression of key genes important for foetal development. Besides, in placenta samples, the genes enriched in the highly methylated regions (HMD) are involved in defence responses. The review that we present here focuses on imprinted genes, but research aiming to delineate the variations that exist at such loci, between placenta from ART and spontaneous pregnancies, would help us to understand how this alternative epigenetic mechanism may contribute to placental remodelling and pregnancy outcomes.

In addition, to date, no study has focused on histone modifications in ART placentas, although higher concentrations of H3K4 trimethylation have been found in mouse blastocysts cultured in vivo than in vitro [183]. Recently, Court and colleagues suggested that placental-specific imprinted loci could be imprinted by an epigenetic mechanism, such as histone modification, independent of germline methylation [30]. Furthermore, other interesting data about miRNAs indicate that they also deserve to be studied in more detail [76, 184]. Studies on combinations of epigenetic factors would also bring additional knowledge about the respective roles of the different epigenetic alterations after ART.

Besides, since gene expression and DNA methylation are sexually dimorphic in male and female placentas it is also important for future epigenetic placental studies to take into account the sex of the foetuses [185]. For example, a study that investigated the epigenetic variations of *ZAC1* in cases of IUGR revealed down-regulated expression in placentas from girls but not boys [133].

Moreover, the link between placental growth and epigenetics was not investigated. It would be interesting to carry out studies comparing placental development during the early steps of foetal life with placental epigenetic results at birth to unravel the sequence of epigenetic events and distinguish between causal changes and the resulting epigenetic landscape.

## Conclusions

Much evidences support the hypothesis that suboptimal trophoblastic invasion due to a disturbed dialogue during the early phases of placentation could potentially explain the higher frequency of adverse pregnancy outcomes, such as miscarriages or preeclampsia, associated with ART. The dialogue between the endometrium and embryo is a crucial step to achieve successful trophoblastic invasion, ensuring a non-complicated pregnancy and the development of healthy offspring. This dialogue seems to be disturbed by ART, either by impairing endometrial receptivity or by modifying the early steps in the epigenetic development of the embryo. But this initially disturbed placentation also gives rise to a smart dialogue between the foetus and placenta, which may bring adaptive responses, notably through epigenetic mechanisms. Indeed, a coordinated group of genes called the imprinted gene network, stimulated by foetal signals, may modify nutrient transfer as well as placental growth and vascularization.

If these mechanisms are overwhelmed, improper maternal-foetal exchanges could occur, potentially leading to abortion or adverse pregnancy outcomes. Fortunately, in most cases, successful adaptation enables normal progress of the pregnancy and healthy offspring. However, these adaptation mechanisms per se could have adverse effects later in life. More research is thus needed to assess the real impact of ART on future health. The better understanding of the placental mechanisms triggered by ART will aim *in fine* to render the ART protocols safer.
